# Stigma and discrimination: barriers to the utilisation of a nutritional program in HIV care services in the Tigray region, Ethiopia

**DOI:** 10.1186/s12889-020-09040-6

**Published:** 2020-06-10

**Authors:** Fisaha Tesfay, Sara Javanparast, Lillian Mwanri, Anna Ziersch

**Affiliations:** 1 Southgate Institute for Health, Society and Equity, Flinders University, Adelaide, Australia; 2 School of Public Health, Mekelle University, Mekelle, Ethiopia; 3 School of Health and Social Development, DeakinUniversity, Melbourne, Australia; 4 College of Medicine and Public Health, Flinders University, Adelaide, Australia; 5 The Discipline of General Practice, Flinders University, Adelaide, Australia

**Keywords:** Stigma, Discrimination, Nutritional programs, Disclosure of HIV status, HIV/AIDS, Ethiopia

## Abstract

**Background:**

In Ethiopia, stigmatising attitudes towards people living with HIV have reduced over time. This is mainly due to improved HIV knowledge and the expansion of access to HIV care and support services. However, HIV stigma and discrimination remain a key challenge and have negative impacts on access to and utilisation of HIV services including nutritional programs in the HIV care setting. A small number of studies have examined the experience of stigma related to nutritional programs, but this is limited. This study explored HIV status disclosure and experience of stigma related to a nutritional program in HIV care settings in Ethiopia and impacts on nutritional program utilisation.

**Methods:**

As part of a larger study, qualitative in-depth interviews were conducted with 20 adults living with HIV, 15 caregivers of children living with HIV and 13 program staff working in the nutritional program in three hospitals in the Tigray region of Northern Ethiopia. Framework thematic analysis was employed to analyse the data and NVivo 11 was used to analyse the qualitative interview data. This study is presented based on the consolidated criteria for reporting of qualitative research (COREQ).

**Results:**

The study found varying levels of positive HIV status disclosure, depending on who the target of disclosure was. Disclosing to family members was reported to be less problematic by most participants. Despite reported benefits of the nutritional program in terms of improving weight and overall health status, adults and caregivers of children living with HIV revealed experiences of stigma and discrimination that were amplified by enrolment to the nutritional program and concerns about unwanted disclosure of positive HIV status. This was due to: a) transporting, consuming and disposing of the nutritional support (Plumpynut/sup) itself, which is associated with HIV in the broader community; b) required increased frequency of visits to HIV services for those enrolled in the nutritional program and associated greater likelihood of being seen there.

**Conclusion:**

There was evidence of concerns about HIV-related stigma and discrimination among individuals enrolled in this program and their family members, which in turn negatively affected the utilisation of the nutritional program and the HIV service more broadly. Stigma and discrimination are a source of health inequity and undermine access to the nutritional program and other HIV services. Nutritional programs in HIV care should include strategies to take these concerns into account by mainstreaming stigma prevention and mitigation activities. Further research should be done to identify innovative ways of facilitating social inclusion to mitigate stigma and improve utilisation.

## Background

Stigma and discrimination associated with human immunodeficiency virus/acquired immunodeficiency syndrome (HIV/AIDS) are an important public health issue that negatively affects health outcomes among people living with HIV [1–4]. HIV/AIDS related stigma and discrimination (hereafter ‘HIV-related stigma’) contributes to mental health issues such as depression, anxiety, emotional and mental distress [3, 5–7]. HIV-related stigma also compromises other health outcomes including physical health and overall quality of life [3, 5–7]. There is also evidence suggesting that HIV-related stigma is one of the key barriers to the utilisation of HIV care services [8, 9].

Stigma is a harmful societal phenomenon enabled by underlying social, political and economic powers labelling and linking differences into negative stereotypes, leading to a separation of “us” from “them,” and finally to status loss and discrimination for those with the trait [10]. Stigma can be perceived, experienced/enacted and internalized [11]. When it comes to HIV-related stigma, perceived stigma is the belief that a person will be discriminated against or judged negatively when their positive HIV status is disclosed, while experienced/enacted stigma refers to the actual events of discrimination experienced by people living with HIV [11, 12]. Internalized stigma refers to the negative self-image felt by those diagnosed with HIV [11, 12].

HIV and AIDS as stigmatizing conditions vary by culture and context, but common among most cultures is the linking of HIV and AIDS to immorality and social deviance, through an association of the disease with groups deemed ‘deviant’ from social norms such as commercial sex workers, drug users and homosexual men [11, 13, 14]. Fear and shame due to concerns of stigma may hinder patients’ disclosure of their positive HIV status, leading to behaviours such as distancing and isolation [15]. Distancing and isolation of the individual living with HIV undermine their interactions with the health service, community, neighbourhood and family members [13, 15]. Thus internalised stigma and discrimination experienced at various levels such as community, institutions and families contribute to social isolation, loss of status and power in the society and health inequalities [13, 16].

### HIV-related stigma in sub-Saharan Africa

HIV-related stigma in resource-limited settings can have significant negative impact on the use of health services, because they deter people living with HIV from accessing HIV care and support [14]. For instance, in addition to sociocultural and economic factors, HIV-related stigma has been reported to be a significant barrier to antiretroviral therapy (ART) medication adherence, contributing to high rates of HIV mortality, especially in Africa [17, 18]. Stigma and discrimination hinder utilisation of HIV services, when patients believe that seeking care would lead to the disclosure of their positive HIV status and subsequent exposure to HIV-related stigma [9, 19]. For people who experience high levels of poverty and poor livelihood [20], HIV-related stigma can also indirectly affect nutritional wellbeing through limiting economic and social opportunities [21, 22].

Studies have reported that the magnitude of HIV-related stigma in sub-Saharan Africa, including Ethiopia, is decreasing because of improved HIV knowledge and the expansion of HIV care and support services [23, 24]. However, HIV-related stigma remains a key barrier to accessing HIV care and services for people living with HIV and AIDS. A greater understanding of the impact of HIV-related stigma is essential for policy and practice to address these issues.

### HIV-related stigma and nutritional programs

Good nutrition is vital for people living with HIV. It enhances the immune system, increases the effectiveness and efficacy of ART, lowers the risk of chronic non-communicable diseases (such as cardiovascular disease and diabetes), and maximises quality of life [25, 26]. Likewise, the impact of poor nutrition on people living with HIV is significant. Due to the fact that HIV and AIDS and undernutrition are interlinked and widespread in sub-Saharan Africa, nutritional interventions remain a key element of HIV treatment, care and support services in the region [27, 28]. Nutritional care and support programs are designed to improve the nutritional status of adults and children living with HIV and enhance treatment outcomes [29]. Nutritional programs range from nutritional counselling to the provision of micronutrient, macronutrient or fortified micronutrient supplementation [30, 31] and are operational in sub-Saharan Africa, including Ethiopia [32]. Studies conducted in resource-constrained settings and sub-Saharan Africa have identified the role of HIV-related stigma as a key barrier to ART non-adherence [33, 34].

A small number of studies have reported the experience of HIV-related stigma in the context of nutritional programs provided outside and inside health care settings [35–38]. For instance, a study conducted in Kenya by Dibari and colleagues reported HIV-related stigma experienced by people consuming nutritional support products [38]. Another study from Ethiopia by Kebede & Haidar also highlighted that HIV-related stigma was a key reason of people living with HIV for non-adherence to nutritional programs [36]. However, none of these studies have identified the specific mechanisms by which nutritional programs may enhance HIV-related stigma and discrimination. Hence, this study sought to examine the impact of HIV-related stigma on experiences of people living with HIV enrolled in a nutritional program in the Tigray region Ethiopia, and the impacts on program utilisation.

### Stigma a ‘fundamental social cause’ of health inequities

In this study, we conceptualise HIV-related stigma as a fundamental social cause of health inequities. We draw on fundamental social cause theory which is an extension of the basic cause theory by Lieberson, et al. [39] and improved by Link & Phelan [40]. This theory argues that a social cause is a fundamental source of health inequities if it impacts multiple outcomes, affects the health outcome through multiple risk factors, involves resources that are essential to avoid or reduce risk, and the relationship between social causes and health is reproduced over time [41].

Fundamental social cause theory is commonly used to understand the impacts of socioeconomic status on health and its contribution to health inequities [42]. However, HIV-related stigma also meets the criteria for being a source of health inequity [16]. According to social cause theory, stigma negatively influences health through a range of pathways such as psychological and structural pathways [16]. Because of its pervasive nature, stigma disrupts multiple life domains such as resources, social relationships, and coping behaviours, and it is related to multiple disease outcomes through multiple social and psychological mechanisms resulting in health inequities [42].

Social cause theory has been applied in a number of studies related to HIV and health disparities [41, 43, 44]. For instance, Turan et al. 2017 reported that HIV-related stigma had a negative impact on HIV-related health outcomes by influencing adherence and retention in HIV care through affecting interpersonal, psychological factors, mental health, and stress processes [44]. In this way, social cause theory can be applied to help understand the impact of HIV-related stigma in undermining utilisation and contributing to inequitable access to nutritional programs in HIV care settings.

## Methods

### Study design

A cross-sectional in-depth qualitative inquiry was employed to explore the experience of HV-related stigma among adults and caregivers of children living with HIV and enrolled in a nutritional program in Ethiopia.

### Study setting

The Tigray regional state is located in Northern Ethiopia and is one of the nine administrative regional states of Ethiopia. In the Tigray region, at the time of data collection in 2016, according to unpublished reports of the Tigray Regional Health Bureau, 40,000 people had received provider-initiated HIV testing and counselling and 1785 had tested positive up to the end of 2018.

This study was conducted in three selected hospitals in the Tigray region, namely Mekelle, Shul, and Lemlem Karl hospitals. At the time of data collection, 737 HIV positive patients had been nutritionally assessed and found to be clinically undernourished in the three selected hospitals. Out of those found clinically undernourished, 668 were provided with therapeutic or supplementary food. The catchment population in these three hospitals was greater than two million.

Over the study period, all HIV patients enrolled in HIV care were assessed for undernutrition during their regular follow up for the HIV service. According to the program guidelines, those with BMI < 18.5 kg/m^2^ were enrolled into the nutritional programs for 3 months if diagnosed with mild acute undernutrition (BMI < 18.5 kg/m^2^ and 16 > kg/m^2^) and 6 months for those diagnosed with severe acute undernutrition (BMI < 16 kg/m^2^). Those enrolled in the program were provided with nutritional support involving Ready to Use Therapeutic Food (RUTF) or Ready to Use Supplementary Food (RUSF), used to reverse undernutrition among people living with HIV, alongside nutritional counselling. RUTF is a Plumpynut® paste for severe acute undernutrition and RUSF is Plumpy’Sup® paste for moderate and mild acute undernutrition [45, 46]. From this point onwards, RUTF/RUSF are referred to as ‘nutritional support’. The nutritional counselling provided in the nutritional program covered seven general nutrition information components as follows [47]:

1. See a health worker for periodic nutrition assessment (including weight).
2. Eat more and different kinds of foods.
3. Maintain a high level of hygiene and sanitation.
4. Drink plenty of clean, safe (boiled or treated) water.
5. Maintain a healthy lifestyle.
6. Seek early treatment for infections and manage symptoms through diet.
7. Take medicines as advised by your health worker and manage food and drug interactions and side effects.

### Participant recruitment and data collection

A total of 48 one-on-one face to face in-depth interviews were conducted with adults (aged ≥15) (*n* = 20) and caregivers of children living with HIV (*n* = 15) who were currently enrolled or had recently been enrolled in the nutritional program – 6 months in the case of adults and 12 months for caregivers as the potential pool of participants was small. Health providers (*n* = 11) and program managers (*n* = 2) managing the nutritional program who had worked in the program for at least a year (to ensure adequate knowledge of the program and experience of diverse patients) were also interviewed. A proportional number of participants were recruited from each study hospital. These groups of participants were selected purposively because they directly influenced or were involved in the nutritional program (Table 1). Purposive sampling enables the recruitment of study participants with the highest potential to provide rich and diverse information relevant to the research problem [48].

**Table 1:** Participants of in-depth interview by study hospital and recruitment criteria

Study Participants	Recruitment criteria	Number recruited per hospital			Total
		Mekelle	Lemlemkarl	Shul	
Adult HIV patients	Enrolled in the nutritional program for at least one visit prior to the interview or within 6 months of graduation	8	6	6	20
Caregivers of children HIV patients	Child enrolled in the nutritional program for at least one visit prior to the interview or within a year of the child’s graduation	6	5	4	15
Health providers	Have been working in the nutritional program for at least 1 year	5	3	3	11
Program managers	Have good knowledge of the program and worked with the program for at 1 year	N/A	N/A	N/A	2
	Total				48

In qualitative studies, the quality and depth of information is more important than the quantity of participants [49]. While there are no clear and agreed guidelines on the optimum number of participants in a qualitative study, we drew on the common principle of ‘saturation’ of information, where repetition of data occurs during the data collection process [49, 50]. As such, recruitment of study participants ceased when data saturation was attained.

Patients visiting the health service were provided with information about the project and given the opportunity to approach the researcher if interested in participating in an interview. Interviews with adults and caregivers of children living with HIV were conducted at the health facilities during their regular follow up for Antiretroviral Therapy (ART), nutritional and other HIV care and support services. Health providers and program managers were sent an invitation to participate and were interviewed in their workplaces.

Interviews were all conducted in the local Tigrigna language and audio recorded. The duration of interviews ranged from 30 to 66 min. The first author (FT) who is conversant in the local language and culture of the area conducted the interviews. He also transcribed and translated all interviews from Tigrigna to English. Participation in this study was voluntary and participants were informed of the benefits and risks associated with participation and were assured that they could opt out at any time and that participation or otherwise would not affect their access to services.

A semi-structured interview guide was developed in light of the literature and piloted before use and customised to fit the different participant groups. The guide explored experiences of the nutritional program more broadly and included several sections relating to HIV stigma including HIV diagnosis disclosure, experiences and concerns about HIV-related stigma generally as well as in relation to the nutritional program and impacts on use of the program. While the order and wording of questions varied, each of the topics were covered in each interview. Field notes were taken during each interview to note visual cues and body language to assist an understanding of the context and facilitate data analysis and interpretation.

### Trustworthiness and rigor

Rigour and trustworthiness refer to the quality of qualitative evidence [51], and credibility, transferability, consistency, and neutrality of findings [52] are the key parameters to ensure it. Various strategies were used to ensure the rigor and trustworthiness of the findings in this study.

To improve the credibility of evidence, triangulation of study participants was employed by involving adults, caregivers, health providers, and program managers and the recruitment of study participants from different hospitals. Four transcripts were used to establish the coding framework and were coded by all authors to improve coding agreement, reliability and minimize bias, and differences in coding were resolved through discussion. In addition, FT conducted the interviews, translated, and transcribed the data which assisted the analysis, interpretation and also credibility of the findings. The detailed description of the study setting and context of HIV may assist the transferability of the findings into a similar context and it is believed that the nutritional program is similar across Ethiopia. While the interview guide was semi-structured, it was ensured that all questions were covered to assist with consistency. Illustrative quotes presented in the results to support each theme and sub-theme or concept may also help the neutrality of findings. In addition to the interview transcripts, field notes and an event log taken during data collection were also used during data analysis and writeup to contribute to the neutrality or confirmability of the findings.

### Data coding and analysis

An inductive framework thematic approach was employed to analyse the data [53, 54], which is particularly suitable for analysing semi-structured interviews [55, 56]. The analysis in this study involved five steps as recommended by Bryman and Burgess 2002 [53] - familiarisation, identifying a thematic framework, indexing, charting and mapping and interpretation [53].

In the first step familiarisation, which involves translation and transcription of all in-depth interviews to English was done by FT. Data was read and re-read to gain an in-depth understanding of the context, concepts, codes, and potential themes. In the second step, identification of an initial thematic framework was developed using four transcripts. Codes, categories, and themes were constructed in the subsequent analysis. Furthermore, themes and categories were formed to represent concepts around the research questions (how the nutritional program in HIV care enhanced HIV-related stigma) based on recurring concepts shared among participants. In the third step, indexing involved application of the thematic framework to the data. All transcripts of adults, caregiver of children living with HIV, health providers, and program managers were coded in NVivo version 11 (QSR, International) using the framework matrix. In the fourth step, charting or the process of reorganising or sorting data was done. As part of the wider study, which examined the challenges of the nutritional program more broadly, the data was charted against the specific HIV-related stigma themes. In the fifth step, mapping and interpretation was done where the relationship between HIV-related stigma themes and categories were established and cross analysis done between the various participants to examine patterns in relation to demographic characteristics. Direct illustrative quotes were extracted and used to explain and describe themes and sub themes.

## Results

### Characteristics of participants

Adult participants were at different stages of the nutritional program ─ seven were in their second month, five were in their third month, and five were in their fourth month or longer in the nutritional follow up. A further three adult participants had completed their nutritional follow up and recovered from undernutrition. Eleven adult participants were enrolled in the nutritional program for the first time, while nine had previously been enrolled.

Of the 15 caregivers participating in the in-depth interview, all except one were the biological mother of the child living with HIV. Ten reported that their children were in the nutritional program for the first time, while five had been previously enrolled. Regarding the stage of their enrolment, four children were at their second visit and four in their fifth visit and one on their third visit. The remaining seven had just completed the nutritional follow up at the time of interview. Table 2 shows the detailed characteristics of the study participants.

**Table 2:** Characteristics of interview participants

Variables		Adult HIV patients	Caregivers of child HIV patients	Health providers and program managers
Mean age		37.2 + 9.7	36 + 7.3	35.3+ 8
Hospital	Mekelle	8	6	5
	Lemlem Karl	6	5	3
	Shul	6	4	3
	Tigray region health bureau			2
Gender	Male	8	0	6
	Female	12	15	7
Residence	Urban	12	11	13
	Rural	8	4	
Educational status	No education	4	5	
	Primary education	10	5	
	Secondary education	6	5	
	BSc and above			13
Marital status	Single	6	1	4
	Married	6	9	8
	Divorced	5	3	1
	Widowed	3	1	

### Experiences of HIV-related stigma

Fear of HIV status disclosure and subsequent HIV-related stigma was identified as a key concern of participants (adults and caregivers). Participation in the nutritional program was seen as potentially contributing to disclosure of positive HIV status through an increased frequency of visits to the service, and transporting, consuming and disposing of the nutritional support.

### Status disclosure and fear of HIV- related stigma

All participant groups (health providers, adult patients, and caregivers) noted the impact of HIV status disclosure and its relationship with HIV-related stigma. The level of positive HIV status disclosure varied depending on the extent of concern of HIV-related stigma of the individual. Those with high concern about HIV-related stigma had not disclosed to many or even any others, but those with less concern had disclosed their positive HIV status more broadly.

Seven adults (four females and three males) and three caregivers (all females) were very reluctant to disclose their positive HIV status to anyone and expressed a high concern about potential HIV-related stigma related to their positive HIV status and its impact on their life such as rejection and abandonment. One participant presented her fear of disclosure of positive HIV status as follows:

About *the stigma you said, I don’t want anyone to know me [positive HIV status] because there is a stigma. For this reason, I live and act like HIV negative (Adult female, age 20s #15).*

Other participants also expressed similar fears and reluctance to disclose to even close family members:

*Even my elder daughter suspects me and asks if I have HIV but I always convince her that I didn’t have any problem. I didn’t tell her because it could harm her psychologically (Adult female, age 30s #1).*

*All of them [family] live in rural area and they didn’t know about me (positive HIV status). My father, mother, and brothers all are alive and they live in rural areas and they know nothing about me (referring positive HIV status). Nobody knows. I and my health providers knows about my positive HIV status and I am leaving alone (Adult male, age 50s #6).*

*My families (mother and father) didn’t know the positive HIV status of my myself and my children. Because my mother may be shocked if she knows I and my children has HIV. Previously, I was visiting them (referring to her family) rarely but now I have stopped visiting them. I cut my relationship with my family and turn my attention to the health facility only. (Caregiver, age 30s #9).*

In contrast, three other adult females felt that disclosure of positive HIV status to family members had some benefits such as extra care during illness, financial support, and assistance with housework and other duties.

Beyond family members, there was particular reluctance about disclosure of positive HIV status beyond family for four adult females and two caregivers due to a potential HIV-related stigma in their neighbourhood and broader community:

*Four of my brothers know about my positive HIV status. None of my friends know about my positive HIV status. Nobody [neighbours] knows about me (Adult female, age 20s #10).*

*All my brothers know about my child’s positive HIV status. I had my elder brother who lives in Mekelle and he was supporting me but no one knows other than him. (Caregiver, age 40s #4).*

Concerns included gossip and impacts on social relationships were expressed:

*There is also gossip that may run behind and around you. Some also may insult you and harm you emotionally at times of disagreement (Adult female, age 20s #15).*

*I don’t know how the secret escapes. Our neighbours didn’t know about his [referring to her HIV positive son] positive HIV status but my son said to me that children deny him to play with them (Caregiver 30s #9)*

Participants also reported fear of being expelled from their house and denial of sharing toilets, bathrooms, and common utensils if positive HIV status was discovered by house owners or housemates:

*Because in house rent we share a toilet, bathroom, and other associated issues. So, if the house owners know that you are HIV positive and using Plumpynut or ART medication, they could expel you from the house (Adult female, age 30s #4).*

An adult female also reported potential economic impacts of HIV-related stigma:

*“If people know about me, it will have an influence on my work in the market. People didn’t come and buy from you if they know their HIV status. If this happens, it will have to influence me and my children” (Adult female, age 20s #15).*

In terms of negative social consequences of disclosure among the adult participants, more women (seven out of the 12 adult female participants compared to three out of eight male participants) expressed concerns to some extent about negative social consequences or discrimination related to HIV disclosure.

A smaller number of adults and caregivers had disclosed their positive HIV status to a broader community and reported benefits such as getting support from friends, families, and others for their HIV condition. For example, one adult female said:

*I am free with everybody and I didn’t hide myself. Everyone knows about me. So, I have not experienced any problem so far. I didn’t bother too much not to be identified by anybody (Adult female, age 30s #16).*

One caregiver reflected on the changes over time of her family’s experience of HIV-related stigma:

*We started ART 6 years ago. By that time, nobody was coming to our house and everybody was afraid of entering into our home (referring to herself and her HIV positive child). In any social event, no one wants to sit with us (referring to herself). There were many problems. But now I am working like any other person and live like any healthy person going to the market and work as well to other social events. So due to this, there is no such problem currently as it was in the previous (early periods of positive HIV status) (Caregiver, age 40s #13).*

While participants acknowledged improvements in HIV-related stigmatising attitudes towards people living with HIV, generally non-disclosure or limited disclosure of positive HIV status significantly affected the extent to which HIV-related stigma was identified by participants as an issue in relation to use of the nutritional program.

### Nutritional program and HIV-related stigma

There were two key mechanisms by which the nutritional program contributed to HIV-related stigma among those attending the HIV clinics and enrolled in the nutritional program– increased frequency of health service visits and the association of the nutritional support with HIV.

Enrolment in the nutritional program increased the frequency of visits to a health facility to obtain the nutritional support and conduct nutritional follow up and monitoring. To receive ART medications and other HIV services, adults and caregivers visit a health facility once every 3 months. For those enrolled in the nutritional program, frequency of visits needed to increase to monthly for a period of three to 6 months. Health providers reported that this increased frequency of visits was seen by patients as increasing the chance of being seen at the service and hence contributing to the involuntary revealing of positive HIV status:

*“There are also individuals whom they don’t want to come more frequently to the clinic not to be seen by any other person whom they know” (Health provider, #2).*

*“Some say they can’t come every month because distance and even transportation is not a problem, they may be afraid of being seen by other persons as they visit the health facility frequently. They want to take it once for about three months or more” (Health provider #5).*

In addition, in the community nutritional support is highly associated with HIV, particularly for adults. Being seen with the nutritional support in the community was thought to immediately raise suspicion of positive HIV status. This led to challenges in relation to the transportation of the nutritional support, and the consumption and disposal of the empty nutritional support sachets:

*Some people relate it. For example, one of our drink shop client told me that the Plumpynut is given to HIV patients on ART. He told us that it is given to them (referring to people living with HIV) for their ART medication (Caregiver, age 30s #5).*

*So, some patients enrolled in the nutritional program are worried of stigma and discrimination. Because the Plumpynut is seen as a food to be given to HIV patients in the community especially in the community (Health provider, #2)*

The typical packing and bulky size of the RUTF/RUSF nutritional support are easily identifiable and revealing, which made transportation of the nutritional support from the health facility to the patient’s place difficult, as a number of participants highlighted:

*I will not show them because instead of its own carton, I use a different bag to carry it home. So, nobody will identify it. When my neighbours ask me, then I pretend that it is cereal. I also don’t want anyone to know me that I am going to a hospital, to avoid stigma (Adult female, age 20s #15).*

*If some know that you are using the medication and Plumpynut because of your HIV status, there may be problems. People usually hide the Plumpynut during transportation and at their home especially when they live in a house rent (Adult male, age 50s #1)*

Similarly, finding a convenient place to consume the nutritional support in private was a challenge due to the revealing nature of the packaging of the nutritional support and fear of being seen by others who would associate it with HIV, as the following account illustrates:

*Even when I take the Plumpynut, I don’t want to show them (neighbours) at all. I consume and bring the empty sachet back to here (the health facility) because I don’t want to be seen. If there is a guest in my home, I will not use the Plumpynut but if there is no one, then I will use it. When using, I close my door to use my Plumpynut and ART medication. Why I did this is to avoid stigmatization. So, the only option I have is to use it like this (Adult female, age 20s #20 ).*

As alluded to in the above quote, the empty sachets were also seen as potentially identifying, and participants were concerned about being seen when throwing the empty sachets at home or anywhere else due to fear of disclosure of their positive HIV status, as one adult reported:

*My daughter had taken the empty sachet outside and our neighbours asked me from where I had brought it acknowledging that it is good food, but I didn’t respond for the question and I kept quiet (Adult female, age 20s #17).*

For one family, the discovery of one of the children’s HIV positive status in this way had been devastating:

*Yes, it is due to the empty sachet we throw that our neighbours identify us and asked him [referring to her HIV positive son] why he is eating it by that time. So, my neighbours usually don’t allow my children to play with them because they know our positive HIV status as well as I told you, he has refused to go to school because the children in his school don’t allow him to play with them (Caregiver, age 30s #9.*

### Impacts of HIV-related stigma on the nutritional program

HIV-related stigma contributed to the problems of effectiveness of the nutritional program in a range of ways. For instance, fear of HIV-related stigma were found to contribute to poorer effectiveness of the nutritional program, where health providers reported that some participants refused to enrol into the program and 5 adult and 3 caregiver participants reported impacts on their program utilisation. Impacts on participation included refusal of the nutritional support, limited attendance at the service, and selling the nutritional support to exchange for other foods.

Health providers identified that a fear of disclosure and HIV-related stigma lead some people living with HIV to refuse to take the nutritional support:

*They told me that they don’t want to take the Plumpynut and they only want to use their own home-made food. This is because they don’t want to be identified as HIV positive. Because if you have Plumpynut, they are afraid that they may be identified as HIV positive. So, the most important is that the clients are afraid of being identified as HIV positive (Health provider, #6).*

Adults also identified this as an issue amongst HIV patients:

*Previously there was fear by HIV patients being identified and some patients were not using the Plumpynut at all (Adult male, age 40s #7).*

*… others even though they are told to take it, they refuse to do so. The reason for this is the issue you raised it (the Plumpynut box) is big as well everybody knows it. There are individuals who doesn’t take it (the Plumpynut) at all. The reason they fail to take it is because of the issue you raised (stigma and discrimination) but I don’t know about others (Adult male, age 40s #22).*

Health providers likewise identified that some patients also minimised their attendance at the service to reduce the chance of being seen and subsequently identified as HIV positive:

*There are also individuals whom they don’t want to come more frequently to the clinic due to fear of being seen by other persons they know (Health provider, # 2).*

*Some patients see some improvement when they take the nutritional support after starting the second month. Because of this, they prefer to stop and don’t want to come to this clinic monthly. You know coming to this clinic is also one reason patients don’t want to happen frequently because they may be identified or seen by some they know. So, most patients don’t want to come more frequently. They believe that they may be identified as HIV patients by someone and leads to stigma and discrimination (Health provider, #6).*

Selling of the nutritional support in order to purchase other foods due to fear of HIV-related stigma was also identified as negatively influencing the utilisation of the nutritional program in HIV care. A caregiver stated this problem as follows:

*People sell the nutritional support because they are afraid of being identified as HIV patients because of fear of stigma. It is now sold up to 6 Birr [Ethiopian currency] (Caregiver, age 40s #14).*

These issues contributed to poor adherence and utilisation of the nutritional program leading to compromised effectiveness of nutritional program in HIV care settings.

## Discussion

The aim of this study was to explore how HIV-related stigma may influence utilisation of nutritional programs in HIV care settings in Ethiopia and more broadly. While some previous studies reported experiences of HIV-related stigma in relation to nutritional programs in HIV care [36, 38], none had explored the possible mechanisms by which nutritional programs in HIV care may enhance HIV-related stigma. This study sought to address this gap.

In the current study, adults and caregivers (their own and the target child) had generally not disclosed their positive HIV status broadly because of concerns of HIV-related stigma. Disclosure varied by the individual persons’ relationship with the target of disclosure and also extent of anticipated negative consequences. More people had disclosed to their families and close relatives. A recent study from Tigray region of Ethiopia similarly reported that few people disclosed their positive HIV status, beyond telling their spouse, because of fear of HIV-related stigma [57]. Furthermore, a systematic review conducted in low resource settings indicated that fear of HIV-related stigma is the fundamental source of positive HIV status non-disclosure [58]. The current study also found that caregivers were afraid of disclosure of positive HIV status of their child (and often also themselves if they were HIV positive) and its negative impacts for them and their children. Other studies by Holzemer, et al. and Florom, et al. have likewise found HIV-related stigma to be an issue for children living with HIV [59, 60].

Participants in this study acknowledged general improvements in people’s stigmatising attitudes and perceptions towards HIV patients. Reduction in stigmatizing experiences was reported in another study from Ethiopia [61]. Furthermore, a systematic review conducted in sub-Saharan Africa reported improvements in the level of tolerance of people living with HIV [19]. However, discriminatory attitudes in the community are still significantly high in Ethiopia, with the 2016 Ethiopian demographic and health survey report highlighting 48% of women and 35% of men reporting discriminatory attitudes [61]. These attitudes include not buying fresh vegetables from a shopkeeper or vendor if they knew that person is HIV positive or agreement that children living with HIV should not be allowed to attend school with children who were HIV negative [61]. Another study in Mekelle-Ethiopia also reported that HIV-related stigma in relation to people living with HIV were still common and HIV-positive people are often isolated [57]. A further study involving health providers also reported concerns about HIV-related stigma for people living with HIV [62]. The findings of the current study add to this literature, further highlighting stigma as an ongoing issue of serious concern where participants reported felt, enacted and internalised HIV stigma.

While some studies have identified HIV-related stigma as a barrier for HIV treatment (ART) adherence [12, 13, 17], other than the present study, there were no other specific studies on how HIV-related stigma related to nutritional program influenced utilisation. Our study found that nutritional programs in HIV care settings potentially enhanced HIV-related stigma by increasing the frequency of visits to the health facility and due to the nutritional support during transportation, consumption, and disposal.

Enrolment in the nutritional program increased the frequency of visit to the health facility, where participants were afraid of meeting someone they knew, and their positive HIV status being revealed. Hence, participants preferred either to stop attending the nutritional program or skip appointment dates. According to Williams, stigmatised individuals may be reluctant to visit a health facility or be visited by health provider if it is felt that such visit may increase suspicion of positive HIV status in the community or undermine concealment of a discredited identity [9].

The other mechanism that the nutritional program enhanced the risk of HIV status disclosure was due to the widely held view in the community that the nutritional support is related to people living with HIV─ meaning if someone is seen in the community with the nutritional support during transportation, consumption or disposal, it could lead to revealing positive HIV status. Hence, adults for themselves and caregivers for their children were reluctant to use the nutritional support and expressed their fears of being identified while using it. Participants felt that it was more exposing than ART medication because of its bigger size and the longer time to consume it. Another study from Ethiopia also reported a strong association of the nutritional support with HIV in the community [36, 37] and a study from Kenya also reported experience of HIV-related stigma related to the easily identifiable packaging box of the nutritional support [38]. This is similar to studies that identified concerns of disclosure of positive HIV status through other HIV services such as ART medication [63], and its impacts on ART adherence [17, 18, 44].

The ultimate impact of HIV-related stigma on the nutritional program is poorer outcomes such as drop out and non-response to the program. HIV-related stigma and discrimination can lead to poor utilisation of the nutritional program which includes failing to consume the recommended ration and missing appointment dates due to fear of disclosure of positive HIV status, which contributed to default and non-response (not recovering from undernutrition). There is no other external study that examined the impact of HIV-related stigma on the utilisation of the nutritional program but a meta-analysis and systematic review on the impact of HIV-related stigma on HIV outcomes pointed out the negative impacts of HIV-related stigma on a range of health outcomes such as lower levels of adherence to antiretroviral medications and limited access to and usage of health and social services [3].

We examined HIV-related stigma in light of the social cause theory which argues that the fundamental social causes of health inequities are indicated when the social cause impacts multiple outcomes, affects the health outcome via multiple pathways, involves resources that are essential to avoid or reduce risk, and the relationship between social causes and health is reproduced over time [64]. More broadly, HIV-related stigma leads to poor socioeconomic status because people living with HIV have limited life chances due to the negative impact of HIV-related stigma [21, 65] and poorer socioeconomic status leading to inequitable access to health and other social services [66]. HIV-related stigma also affects health through loss of power, declining social status, internalised negative self-image or ‘spoiled’ identity, and stress and structural barriers [16].

Stigma in this study was a fundamental barrier to utilisation of the nutritional program in HIV care settings where the nutritional program in HIV care potentially contributes to HIV-related stigma through facilitating unwanted disclosure of positive HIV status. HIV-related stigma is one of the key factors that negatively influences health seeking behaviour of individuals [67]. In addition, HIV is more prevalent among certain groups such as commercial sex workers, the poor, and racial and ethnic minorities [67, 68], and as such can contribute to health inequalities by creating differences in access to health service and resources to reduce the risk of disease [69, 70]. HIV-related stigma is a source of health disparity for many health outcomes other than the nutritional program which is consistent with the fundamental cause theory [71]. As such addressing HIV-related stigma is crucial in the fight to improve health equity.

### Limitations of the study

The study drew on multiple perspectives from three different sites but was not without limitations. The study is qualitative and may not be more broadly generalisable. Some original meaning of the transcripts may be lost during translation, though substantial effort was made to maintain the originality of findings through verbatim translation, transcription and a translation accuracy test. Having a man interviewing women may also be a source of concern but the interviewer (FT) was particularly mindful of this and having grown up in the area and understanding the local culture was particularly helpful in considering gendered communication during the interview. The interview was conducted in the health facility which may have made participants more reluctant to fully express their views about HIV-related stigma related to the program due to fear of service denial. However, participants were continuously reassured that their participation or otherwise would not affect the service they receive both in the nutritional program and other HIV services.

## Conclusion

HIV-related stigma is an important public health problem - as stated by Kenneth Cole (an international goodwill ambassador): “It has been said that HIV-related stigma has killed more people than the HIV virus,” [72]. There is evidence that enrolment in the nutritional program was associated with increased concern about HIV-related stigma. This negatively affected the utilisation of the HIV service more broadly and the nutritional program more specifically, potentially contributing to health disparities. Thus, nutritional programs in HIV care should include strategies that take these concerns into account. This should include the incorporation of HIV-related stigma prevention strategies such as innovative methods of social inclusion and involvement of community support groups. Studies have also highlighted the potential role of religious leaders in the fight against HIV-related stigma [73, 74] so involving these leaders is important. The packaging of the nutritional support should be modified into non-discriminatory colour, type and to resemble packaging used in the community. In addition, people should be given more rations to minimise the frequency of visit, though this study did not determine the optimal ration or frequency of visit acceptable for adults and caregivers of people living with HIV. As such further research should be done to examine the effectiveness of the stigma-reduction strategies.

## Data Availability

The datasets used and/or analysed during the current study are not available due to the highly sensitive nature of the data and ethical concerns about confidentiality. In addition, the researchers assured the ethical review committees and study participants that data would not be shared with a third party.
